# Dietary Vitamin D3 Supplements Reduce Demyelination in the Cuprizone Model

**DOI:** 10.1371/journal.pone.0026262

**Published:** 2011-10-20

**Authors:** Stig Wergeland, Øivind Torkildsen, Kjell-Morten Myhr, Lage Aksnes, Sverre Jarl Mørk, Lars Bø

**Affiliations:** 1 Department of Neurology, Norwegian Multiple Sclerosis Competence Centre and KG Jebsen Centre for Multiple Sclerosis Research, Haukeland University Hospital, Bergen, Norway; 2 Department of Clinical Medicine, University of Bergen, Bergen, Norway; 3 Department of Pathology, Haukeland University Hospital, Bergen, Norway; Innsbruck Medical University, Austria

## Abstract

Vitamin D is emerging as a probably important environmental risk factor in multiple sclerosis, affecting both susceptibility and disease progression. It is not known to what extent this effect is due to a modulation of peripheral lymphocyte function, or to intrathecal effects of vitamin D. We investigated the effect of dietary vitamin D3 content on de/remyelination in the cuprizone model, which is a well established toxic model of demyelination, with no associated lymphocyte infiltration. The mice received diets either deficient of (<50 IU/kg), or supplemented with low (500 IU/kg), high (6200 IU/kg) or very high (12500 IU/kg) amounts of vit D3. Cuprizone (0.2%) was added to the diet for six weeks, starting two weeks after onset of the experimental diets. Mouse brain tissue was histopathologically evaluated for myelin and oligodendrocyte loss, microglia/macrophage activation, and lymphocyte infiltration after six weeks of cuprizone exposure, and two weeks after discontinuation of cuprizone exposure. High and very high doses of vitamin D3 significantly reduced the extent of white matter demyelination (p = 0.004) and attenuated microglia activation (p = 0.001). No differences in the density of oligodendrocytes were observed between the diet groups. Two weeks after discontinuation of cuprizone exposure, remyelination was only detectable in the white matter of mice receiving diets deficient of or with low vitamin D3 content. In conclusion, high dietary doses of vitamin D3 reduce the extent of demyelination, and attenuate microglia activation and macrophage infiltration in a toxic model of demyelination, independent of lymphocyte infiltration.

## Introduction

Multiple sclerosis (MS) is a chronic inflammatory demyelinating disease of the central nervous system (CNS), affecting mainly young adults. The prevalence of MS is increasing; it seems to be caused by a complex interplay between environmental and genetic risk factors in susceptible individuals [1], [2]. Vitamin D_3_ (cholecalciferol, vit. D_3_) is suggested as an environmental factor with disease modifying functions [3], [4]. Longitudinal epidemiological studies have shown an increased risk of MS in persons with low s-vitamin D_3_ levels [5]–[8], and low dietary intake and less sunlight exposure are associated with an increased risk of the disease [8]–[12]. Low serum levels of vitamin D_3_ has been found in relation to MS relapses [13], and high serum levels of vitamin D_3_ in MS-patients seem to reduce the hazard ratio for new relapses in a linear relationship [14].

In experimental autoimmune encephalomyelitis (EAE), a T-cell dependent animal model for MS, the activated form of vitamin D_3_, 1,25-dihydroxy-vitamin D_3_ (1,25(OH)_2_D_3_) is highly efficient in both disease prevention and treatment [15]. The clinical effects of vitamin D_3_ are mediated via the vitamin D receptor (VDR), which is widely distributed both on T-lymphocytes and in the CNS. The VDR may be upregulated in activated and 1,25(OH)_2_D_3_ stimulated T-cells [16]. 1,25(OH)_2_-Vit D_3_ stimulation inhibits transcription and secretion of pro-inflammatory cytokines, and skews CD4+ T-lymphocytes toward a Th2 cytokine profile [17], [18].

The mechanisms for how vitamin D influences disease risk and disease progression are, however, poorly understood. Vitamin D status is also hypothesized to influence the risk and progression of other, neurodegenerative disorders like Parkinsons disease [19]–[21] and Alzheimers disease [19], [22], in addition to schizophrenia [23], [24]. This suggests that vitamin D has a role in the development and function of the CNS beyond a modulation of T cell functions [25].

In the CNS of healthy individuals, VDR is expressed in both neuronal- and glial cells of the gray matter, but scarcely in the white matter [26]. In addition, the enzyme converting vitamin D_3_ into its biologically active form, 1,25(OH)_2_ D_3_, 1α-hydroxylase, is widely expressed in neuronal and glial cells in the human CNS [26]–[28].

The cuprizone model of de- and remyelination is a non T-cell dependent model of toxic de- and remyelination [29], [30]. The copper chelator cuprizone induces selective oligodendrocyte death, followed by myelin disruption, astrogliosis and microglia- and macrophage activation. The model allows investigation of the effects of vitamin D_3_ on oligodendrocyte/myelin loss and regeneration independent of a modulation of T lymphocyte functions.

In this study we investigated the effect of the precursor form, unhydroxylated vitamin D_3,_ the form physiologically provided by dietary intake or UVB exposure in humans. We aimed to investigate how different vitamin D_3_ levels in the diet influence oligodendrocyte loss, demyelination and remyelination in the cuprizone model for MS.

## Materials and Methods

### Mice

72 five-week-old female C57Bl/6 mice (Tacomic, Tornbjerg, Denmark), with a mean weight of 20.4 g±1.1 g were used for the experiment. They were housed six together in Macrolon IVC-II cages (Scanbur, Karlslunde, Denmark) in standard laboratory conditions; light/dark cycles of 12/12 hours, cage temperature of 22.5±1°C, relative humidity of 52±5% and 75 air changes per hour. Cage maintenance was performed once weekly, and the animals were handled by the same individuals at all times. The animals were weighed twice weekly. The experiment was conducted in strict accordance with the Federation of European Laboratory Animal Science Associations recommendations, and the protocol was approved by the Norwegian Animal Research Authority (permit #2009-1767).

### Vitamin D3 diets

Four diets, only differing in the content of vitamin D_3_ were custom made by a commercial manufacturer (Altromin GmbH, Lage, Germany). The vitamin D_3_ content was 1) <50 IU/kg (e.g. deficient), 2) 500 IU/kg, 3) 6200 IU/kg and 4) 12500 IU/kg, respectively. The content was verified by HPLC in an independent laboratory (Norwegian Institute for Nutrition and Seafood Research, Bergen, Norway). The different vitamin D_3_ doses were chosen to reflect relevant human dietary doses, ranging from deficiency to the doses used in intervention studies in MS. Assuming a daily intake of 5 g of chow per mouse per day, allometric conversion as described earlier [31] gives estimated human equivalent doses of 1) <76 IU/day, 2) 760 IU/day, 3) 9425 IU/day and 4) 19003 IU/day for the different diets.

### Experimental groups and cuprizone administration

After one week acclimatization, the mice (n = 72) were randomised to one of the four experimental diets. Two weeks after randomisation, when the mice were eight weeks of age, 2/3 of the animals in each diet group (n = 12) were randomised to cuprizone exposure, while the remaining six animals in each diet group served as healthy controls. To induce demyelination, cuprizone (bis-cyclohexanone-oxaldihydrazone, Sigma-Aldrich, St. Louis, MO, USA) 0.2% (w/w) was added to the milled mouse chow. The mice had ad libitum access to chow and tap water during the whole experimental period.

### S-25(OH)-vitamin D_3_ and s-calcium analysis

Serum was collected at four time points during the study: 1) before randomisation to the experimental diets, 2) After the two-week wash-in period, before cuprizone exposure, 3) after six weeks of cuprizone exposure, and 4) after two weeks recovery after ending cuprizone exposure.The 25(OH)-vitamin D_3_ analysis was performed according to a modified version of a method described previously [32]. Briefly, 25 µl serum samples were spiked with 26,27-dexadeuterium-25-OH-Vitamin D_3_ (Synthetica AS, Oslo, Norway) as internal standard and extracted with methanol and n-hexane. The n-hexane phase was collected, evaporated to dryness and ejected into a reverse-phase high performance liquid chromatography system. Elution of 25-OH-vit D_3_ was performed with methanol/water (88∶12, v/v, with 0.1% formic acid) and the eluate was monitored by a LC/MS-detector (LC/MSD SL, Agilent Technology INC, CA 95051, USA) equipped with a multimode ion-source. 25(OH)-vit. D_3_ and internal standard were monitored at 395.0 and 407.3 m/z , respectively, in the APCI positive mode. The mean recovery of 25(OH)-vitamin D_3_ was 77.2% (SD 3.9%) and the interassay variation was 4.9%, with a detection limit <4 nmol/l. Serum calcium was analysed using Calsium AS FS (DiaSys Diagnostic Systems GmbH, Holtzheim, Germany) [33].

### Histopathology

Mice were asphyxiated with CO_2_, the brains removed and fixated in 4% paraformaldehyde for at least 7 days, then paraffin embedded. All analyses were performed on 7 µm sections ±1 mm from the bregma [34]. Sections were stained with Luxol Fast Blue (LFB) and hematoxyline and eosine (HE). For immunohistochemistry, the sections were dewaxed and rehydrated before antigen retrieval in either citrate buffer (pH 6.2) or Tris-EDTA buffer (pH 9.1). Primary antibodies and their incubation times and temperatures are given in Table 1. Sections were blocked with peroxidase blocking solution (Dako, Glostrup, Denmark), and visualized with EnVision 3.3 - diaminobenzidine (1∶50; 10 min at room temperature (RT); (Dako, Glostrup, Denmark). The tissue sections were counterstained with hematoxylin. For each antibody, omission of the primary antibody served as negative control. Appropriate positive controls were included in each staining procedure. For proteolipid protein (PLP), Neurite outgrowth inhibitor A (NOGO-A), and Mac-3, normal brain tissue from the healthy controls served as positive controls. For the T-lymphocyte marker CD3, sections from tonsils served as positive controls.

**Table 1:** Antibodies used for immunohistochemistry.

Target antigen	Species/isotype	Working dilution	Incubation time/Temperature	Antigen demasking*	Source
**Proteolipid Protein (PLP)**	Mouse IgG2a	1∶1000	24 h/4°C	Citrate	Serotec, Oxford (UK)
**Neurite Outgrowth Inhibitor Protein A (NOGO-A)**	Rabbit polyclonal	1∶1000	1 h/RT	Citrate	Chemicon, Temecula (CA), USA
**MAC-3**	Rat IgG1, K	1∶200	24 h/4°C	Citrate	BD Biosciences, Franklin Lakes (NJ), USA
**CD3**	Rabbit polyclonal	1∶4000	1 h/RT	Tris-EDTA	Sigma-Aldrich, St. Louis (MO), USA

RT = room temperature.

*Demasking by boiling (micorowaving) sections in the respective buffer for 15 min.

### Characterisation of brain tissue

All sections were evaluated by two blinded observers, using light microscopy (Leica DMLe, Wetzlar, Germany). For quantification of myelin, sections stained with LFB and PLP were scored semiquantitatively for myelin loss in the midline of corpus callosum using a scale ranging from 0 (complete loss of myelin) to 3 (complete myelination), as described by Lindner and colleagues [35]. In tissue sections immunostained for PLP, the midline of the corpus callosum was photographed with identical exposure settings at 40× magnification (Leica DMLe with Leica DC300 camera). Greyscale images were thresholded in order to avoid quantitative registration of low-intensity background staining. The area of PLP immunopositivity in each image was determined using Image processing and analysis in Java (U. S. National Institutes of Health; Bethesda 2009), and expressed as the percentage of pixels, or relative area, in each image with an intensity within the threshold values. The number of oligodendrocytes (NOGO-A immunopositive cells) [36], microglia, macrophages (Mac-3 immunopositive cells), and T-lymphocytes (CD3-positive cells) was counted in an area of 0,0625 mm^2^ in the midline of the corpus callosum [34], using an ocular morphometric grid.

### Statistical methods

One-way analysis of variance (ANOVA) was used to analyse diet effects. Fishers' least significant difference method was used as post hoc analysis where applicable. Six cuprizone exposed mice and 6 control mice were included in the data analysis for each diet group. All data are presented as arithmetic mean +/−1 standard deviation. Statistical analyses were performed using PASW Statistics 18.0 (IBM; Chicago, 2010).

## Results

### The dietary vitamin D_3_ content is reflected in serum

**Figure 1:**
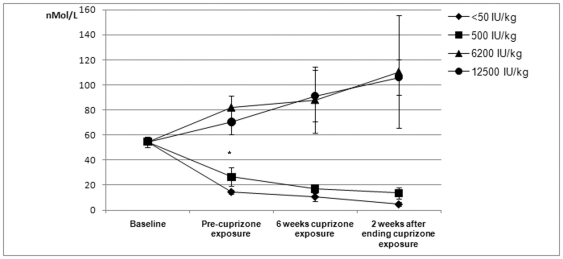
Serum levels of the vitamin D_3_ metabolite 25-OH-vit. D3. After introduction of the experimental diets the serum levels of mice fed a diet with high vit. D_3_ content (6200 or 12500 IU/kg) are significantly higher than in mice fed a diet containing a very low (<50 IU/kg) or low (500 IU/kg) amounts of vit. D_3_ (* p<0.0005). This difference persisted throughout the study period. However, there were no significant differences between the two high-content groups (p = 0.70) or between the two low-content groups (p = 0.46).

The analysis of the vitamin D_3_ metabolite 25-OH-D_3_ in serum samples obtained at different time points during the study revealed that the vitamin D_3_ content in the diet was reflected in the serum of the mice (Figure 1). Prior to the experimental period, all animals were fed a standard rodent diet (Rat and mouse No. I″ maintenance diet from Scanbur, Special Diets Services, Karlslunde, Denmark), containing 900 IU/kg of vitamin D_3_. The mean baseline s-25-OH-vit. D_3_ level was 54.5±4.2 nmol/L. After two weeks on the experimental diet, the serum levels raised or fell significantly in accordance with the vitamin D_3_ content in the diet (p<0.0005). There were no significant differences in the 25-OH-D_3_ serum levels between the cuprizone-exposed mice and control mice (p = 0.732). An increase in serum calcium was observed in all mice during the study period, less in the cuprizone exposed mice than in the controls (p = 0.001). There was no difference in the calcium levels of cuprizone exposed mice between the different diet groups (Figure S1). There was no significant difference between the diet groups with regards to baseline body weight or weight changes (Figure S2).

### High content of vitamin D_3_ in the diet reduces demyelination

**Figure 2:**
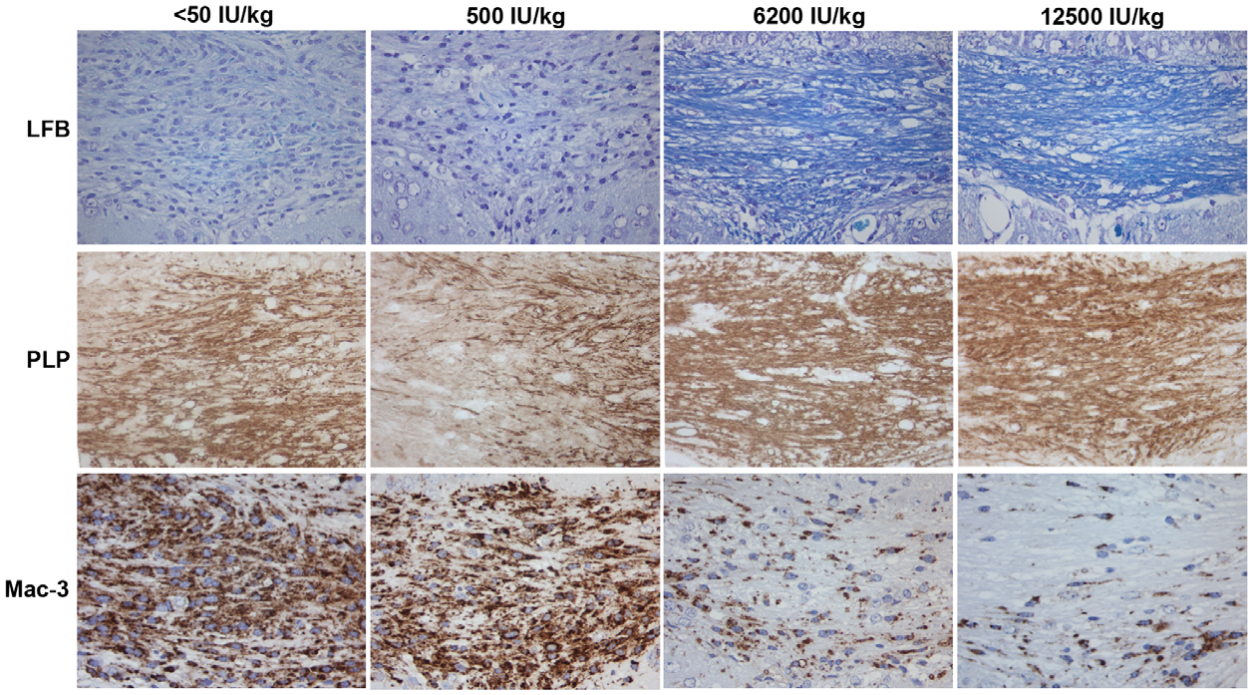
Demyelination and microglia/macrophage activation in the midline of corpus callosum after six weeks of cuprizone exposure in the different diet groups. The mice receiving a diet deficient (<50 IU/kg) or low (500 IU/kg) in vitamin D_3_ content are extensively demyelinated, and the density of activated microglia and macrophages is high. The mice receiving a diet with higher vitamin D_3_ content (6200 or 12500 IU/kg), are significantly less demyelinated (p = 0.004), and the density of activated microglia and macrophages is significantly lower (p = 0.001). All images at 40×.

**Figure 3:**
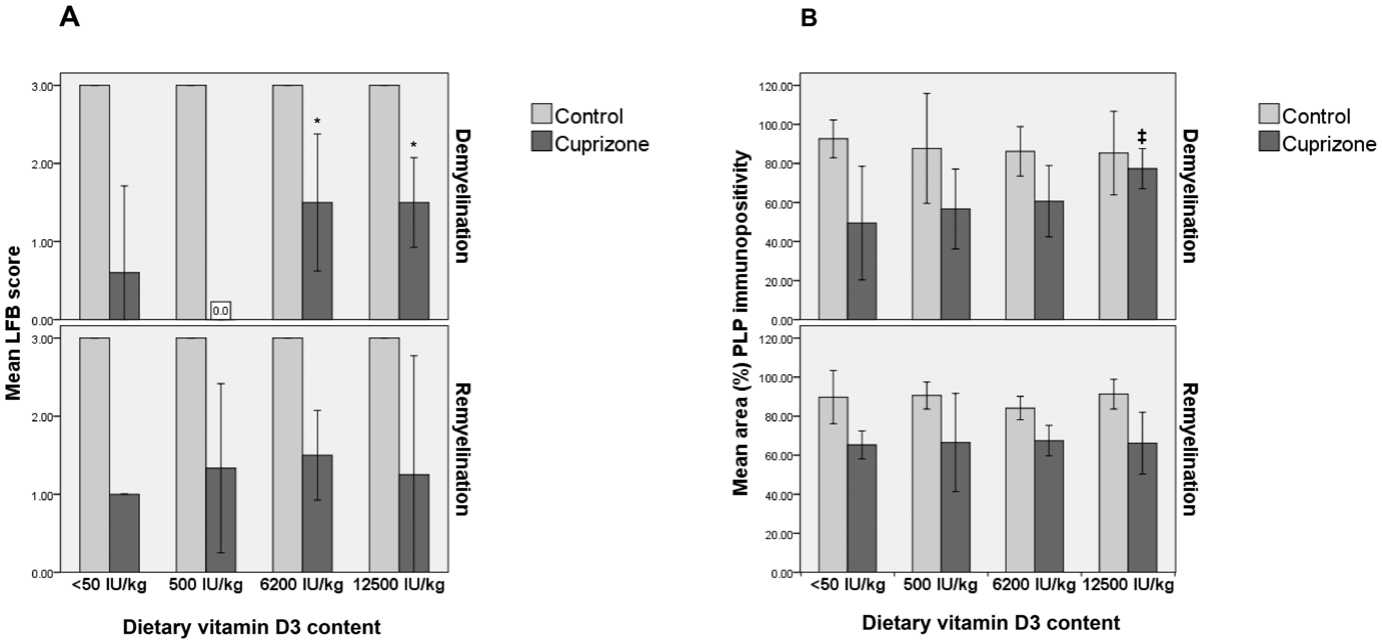
LFB and PLP scores. A: Semiquantitative scores of sections histochemically stained for LFB. A score of 3 indicates normal or full myelination, while a score of 0 indicates complete demyelination. Top panel represents the scores after six weeks of cuprizone exposure, bottom panel two weeks after discontinuation of the cuprizone exposure. Bars represent mean score, error bars represents ±1 SD. * p = 0.004. B: Relative area immunopositive for PLP in the midline of corpus callosum. **Top panel** represents the scores after six weeks of cuprizone exposure, **bottom panel** two weeks after discontinuation of the cuprizone exposure. Bars represent mean score, error bars represents ±1 SD. ‡ p = 0.02.

Significant myelin loss was observed in the corpus callosum after six weeks of cuprizone exposure in all diet groups compared to the control mice (p<0.0005). There were no differences in myelin status between the control mice in the different diet groups. The two cuprizone-exposed groups receiving the highest vitamin D_3_ content, 6200 and 12500 IU/kg, had significantly less demyelination in the corpus callosum than the groups receiving <50 or 500 IU/kg (p = 0.004) as determined in LFB-stained sections (Figures 2, 3). There were no differences in the extent of demyelination between the groups receiving 6200 IU/kg and 12500 IU/kg, or between the group receiving <50 IU/kg and 500 IU/kg. In tissue sections immunostained for PLP, a similar pattern was observed, as the mice receiving 12500 IU/kg vitamin D_3_ in the diet had significantly less myelin loss compared to the vitamin D3 deficient diet (p = 0.02).

In all diet groups, an extensive loss of NOGO-A immunopositive cells was observed after six weeks of cuprizone exposure (Table 2). There were no significant differences in the degree of oligodendrocyte loss (p = 0.18) between the diet groups. Two weeks after discontinuation of the cuprizone exposure, the number of NOGO-A immunopositive cells were similar to the control mice in all diet groups.

**Table 2:** Density of NOGO-A immunopositive mature oligodendrocytes and Mac-3 immunopositive microglia and macrophages in the midline of corpus callosum.

		NOGO-A			Mac-3		
		Control	6 weeks cuprizone exposure	2 weeks after ending cuprizone exposure	Control	6 weeks cuprizone exposure	2 weeks after ending cuprizone exposure
**Diet**	**<50 IU/kg**	61.0 (11.8)	14.8 (10.0)*	71.0 (7.8)	3.2 (2.3)	249.7 (116.2)	69.6 (19.0)
	**500 IU/kg**	57.8 (12.4)	6.0 (5.8)*	60.2 (15.9)	1.2 (1.0)	270.0 (47.9)	45.0 (20.4)
	**6200 IU/kg**	62.3 (17.3)	16.2 (6.2)*	72.7 (9.9)	2.3 (2.3)	95.3 (85.4)**	32.2 (10.4)
	**12500 IU/kg**	69.5 (12.0)	11.6 (8.0)*	62.3 (7.9)	2.8 (3.0)	107.1 (53.1)**	71.5 (34.6)

Cell counts are provided as mean (± SD) number of cells per 0.0625 mm^2^.

*p<0.005.

**p = 0.001.

### Effects on remyelination

**Figure 4:**
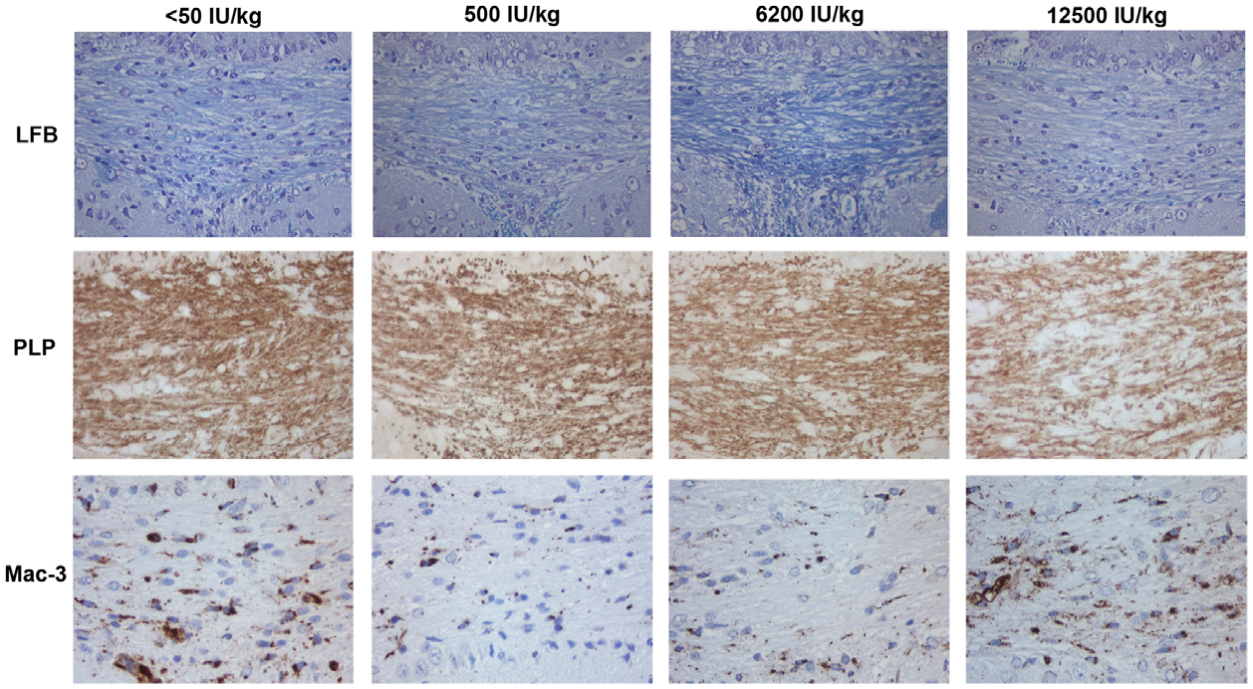
Remyelination and microglia/macrophage activation in the midline of corpus callosum two weeks after discontinuation of cuprizone exposure in the different diet groups. Only the mice given a diet containing <50 or 500 IU/kg of vitamin D_3_ had an increase in LFB semiquantitative scores compared to at the discontinuation of the cuprizone exposure (p = 0.003). There is no significant difference in the mean LFB score between the diet groups (p = 0.74), or in the PLP-immunopositive area (p = 0.99). Microglia/macrophage infiltration is reduced two weeks after ending cuprizone exposure compared to after 6 weeks cuprizone exposure. The reduction in Mac-3 immunopositive cells is most evident in the groups fed the diets with the lower (<50 or 500 IU/kg) vitamin D_3_ content (<50 IU/kg: p = 0.018, 500 IU/kg: p = 0.014). All images at 40×.

Two weeks after discontinuation of the cuprizone exposure, there was no significant difference in myelin status between the cuprizone exposed diet groups (p = 0.74). In the groups fed a vitamin D3 -low (500 IU/kg) or -deficient (<50 IU/kg) diet the myelin status, as evaluated by LFB staining, had improved. This was however not confirmed in tissue sections stained for PLP (Figures 3, 4).

### Dietary vitamin D3 attenuates microglia activation and macrophage infiltration

After six weeks of cuprizone exposure, microglia activation and macrophage infiltration were observed in all diet groups compared to the control groups, as measured by immunohistochemical staining for Mac-3. However, in the groups fed a high vitamin D_3_-diet (6200 or 12500 IU/kg), the number of Mac-3 immunopositive cells were significantly lower than in the groups fed a diet deficient of vitamin D_3_ (<50 IU/kg), or with a low (500 IU/kg) vitamin D_3_ content (p = 0.001) (Table 2, Figure 2). There were no significant differences in the Mac-3 immunopositive cell counts between the mice fed 6200 IU/kg and 12 500 IU/kg (p = 0.795), or between the mice fed <50 IU/kg and 500 IU/kg (p = 0.682). Two weeks after discontinuation of the cuprizone treatment, there was a significant reduction in the Mac-3 immunopositive cell density in the groups fed <50 IU/kg and 500 IU/kg (p = 0.018 and p = 0.014, respectively). A reduction was also observed in the groups fed 6200 IU/kg and 12500 IU/kg, this reduction was not statistically significant (p = 0.054 and 0.344) (Table 2, Figure 4).

### T-lymphocyte infiltration is not present in any of the intervention groups

The extent of T-lymphocyte infiltration in the corpus callosum was studied by immunohistochemical staining for the pan-T-lymphocyte marker CD3. There was no significantly increased infiltration of CD3 immunopositive cells in the cuprizone exposed mice compared to in the controls (data not shown). Only single immunopositive cells contained within blood vessel walls were observed in both groups.

## Discussion

In this study, it was found that vitamin D_3_, provided as a dietary supplement, protected against cuprizone-induced demyelination, and reduced microglia and macrophage activation. After six weeks of cuprizone exposure, mice fed a diet with a high vitamin D_3_ content had significantly less myelin loss in the midline of corpus callosum as evaluated by LFB staining and PLP immunohistochemistry, both validated markers of myelin status [35]. The results were in line with the results of epidemiological studies that have reported correlations between high vitamin D levels and a reduced risk of MS [5], [6] and as well as reduced relapse activity among MS patients [14].

Previous studies of the effects of vitamin D_3_ and 1,25(OH)_2_D_3_ in demyelinating animal models have focussed mainly on the inflammatory, T-cell driven EAE. Our results suggest that vitamin D_3_ also has myelin-protective effects independent of T-lymphocyte activation and infiltration. We hypothesise that the reduced degree of demyelination observed in the groups receiving the highest doses of vitamin D_3_ supplements could be due to reduced microglia activation and macrophage infiltration.

High vitamin D_3_ content in the diet was significantly associated with attenuated white matter microglia-activation/macrophage infiltration during oligodendrocyte death and demyelination. It has been demonstrated that the active metabolite 1,25(OH)_2_D_3_ has immunomodulatory effects by inhibiting differentiation of dendritic cells, and de-sensitize them to maturing stimuli [37]. Macrophages and monocytes express both the vitamin D receptor (VDR) and 1α-hydroxylase, and VDR is upregulated by 1,25(OH)_2_D_3_ stimulation in vitro [16], [27]. In addition, the enzyme 24-hydroxylase, which degrades 1,25(OH)_2_D_3_ is downregulated in activated macrophages [38], [39]. No direct effects of un-hydroxylated vitamin D_3_ on macrophage and microglia activation and maturation have been demonstrated previously, but the present results suggest that the myelin protective effect of vitamin D is dependent of a modulation of macrophage/microglial function.

No difference in the degree of oligodendrocyte loss was detected after 6 weeks of cuprizone exposure. In the cuprizone model, signs of oligodendrocyte death can be observed as early as after one week of cuprizone exposure [40]. Differences in the survival time of the oligodendrocytes could explain the observed discrepancy between an equal loss of oligodendrocytes but different degree of demyelination observed after six weeks of cuprizone exposure. It is possible that diets with a low/deficient vitamin D_3_ content induced a rapid and extensive loss of oligodendrocytes in cuprizone-exposed mice, while diets with a high/very high vitamin D_3_ content may have induced oligodendrocyte death only after several weeks of cuprizone exposure. In further studies, examining the differences in oligodendrocyte density after two or three weeks of cuprizone exposure would address this question.

The serum 25-OH-D_3_ levels of mice fed diets with a high- or very high content of vitamin D_3_ increased during the whole study period of 10 weeks. The serum levels reflected the >10-fold difference in vitamin D_3_ content between the two low-dose and the two high-dose diets, but did not reflect the 2-fold difference between the two low-dose and between the two high-dose diets. Between these high dose or low dose treatment groups no differences were detected for any of the outcome parameters. This could be due to that cuprizone exposure was initiated before serum vitamin D_3_ levels were saturated. The serum profile indicates that 10 weeks may not be sufficient time to achieve saturated serum levels of 25-OH-D_3_, and preferably the pre-treatment period prior to cuprizone exposure should have been longer. However, the sensitivity of C57Bl/6 mice to cuprizone wanes with increasing age [41], limiting the time period available for pre-treatment.

The results could theoretically be influenced by an interaction between cuprizone and vitamin D_3_, affecting the uptake and action of either cuprizone or vitamin D_3_. However, the serum 25-OH-vit D_3_ levels of the mice did not support this, making an effect on vitamin D_3_ absorption less likely. Apart from the vitamin D_3_ content, the diets did not differ with regards to any other constituents.

To our knowledge, this is the first study to investigate the potentially myelin-protective effects of vitamin D_3_ in the cuprizone model. We have previously shown that a salmon based diet with high ω-3 polyunsaturated fatty acids (PUFA) levels proved superior to a cod liver oil based diet, with higher levels of both ω-3 PUFAs and vitamin D_3_ [42], [43] This may suggest a complex interplay between the dietary constituents.

A high dietary vitamin D_3_ content did not improve the degree or rate of remyelination, as evaluated by LFB or PLP staining. Two weeks after ending cuprizone exposure, remyelination was only observed in low/deficient vitamin D_3_ content diet groups. These groups were extensively demyelinated at the end of cuprizone exposure. This may be due to a higher sensitivity of detecting remyelination in tissue completely or completely demyelinated at earlier time points. Further, the attenuated microglia activation and macrophage infiltration observed in the mice fed a diet with high vitamin D content could influence the degree of remyelination. Microglia activation is a physiological response to CNS injury, facilitating repair mechanisms [44]. Activated microglia and macrophages release growth factors and inflammatory cytokines with a neurotrophic effect, stimulating migration and differentiation of oligodendrocyte precursor cells [45]. Significant remyelination was detected in LFB-stained tissue sections, indicating a high sensitivity of LFB for the detection of partial changes in myelin density. This is supported by the sensitivity of LFB for the detection of partial demyelination in diffuse white matter changes in MS [46].

Modulatory effects on EAE by vitamin D_3_ has only been shown in female mice with intact ovaries, indicating an important correlation between the regulation of vitamin D and oestrogen – a correlation which is well known in humans [47]–[51]. In our study, only female mice were used. It would be of interest to study the effect of gender on thepresent results, however, cuprizone exposure has been shown to disrupt the oestrous cycle [52], thus making studies on gender differences less reliable.

The effect of 1,25 (OH)_2_ – Vit D_3_ in EAE has been shown to be associated with a rise in serum calcium levels [53]. In this study, the dietary calsium content was similar in all diets, and no difference in the serum calcium levels was detected between the diet groups. This suggests that the effect of vitamin D_3_ in the cuprizone model is not dependent on elevation of calcium levels.

In conclusion, vitamin D_3_ provided as a dietary supplement attenuate demyelination and microglia activation/macrophage infiltration in a model of demyelination, independent of CNS leukocyte infiltration.

## Supporting Information

Figure S1 **Serum calcium levels at different time points of the study.** **Left panel:** s-Ca by diet. 2 weeks after ending cuprizone exposure, a significant increase in s-Ca levels is observed (**p<0.0005), but not during the cuprizone exposure period. No difference in s-Ca levels are observed between the different diet groups at any time point. **Right panel:** s-Ca in cuprizone exposed mice vs controls. After 6 weeks cuprizone exposure, the cuprizone exposed mice has significantly lower s-Ca serum levels than in controls (* p = 0.001). Error bars represent 1 SD.
(TIF)

Figure S2 **Weight development during the experimental period.** Cuprizone exposed mice experienced weight loss compared to the animals in control groups. No differences between the diet groups were detected; neither for cuprizone exposed nor control animals. The control animals are pooled in one group. Error bars represent 1 SD.
(TIF)
