# Supplementary figures and images for: Dietary Vitamin D3 Supplements Reduce Demyelination in the Cuprizone Model
Source: PLoS One. 2011 Oct 20;6(10):e26262. doi: 10.1371/journal.pone.0026262 (PMC3197632; doi:10.1371/journal.pone.0026262)

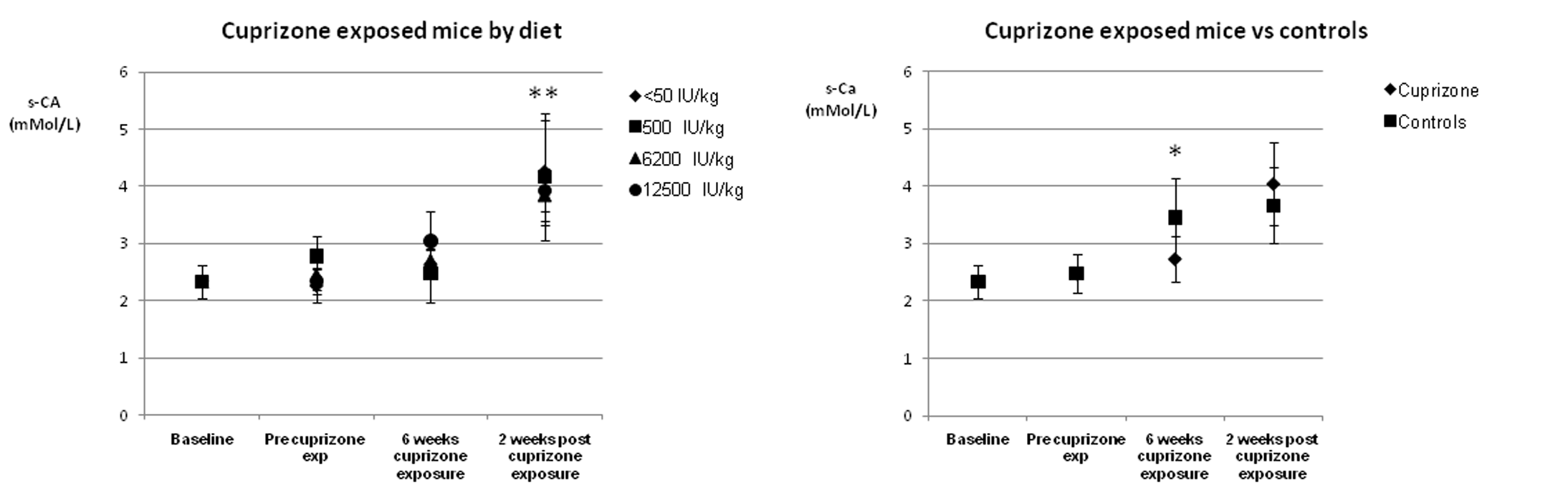

Supplement: Figure S1 — Serum calcium levels at different time points of the study. Left panel: s-Ca by diet. 2 weeks after ending cuprizone exposure, a significant increase in s-Ca levels is observed (**p<0.0005), but not during the cuprizone exposure period. No difference in s-Ca levels are observed between the different diet groups at any time point. Right panel: s-Ca in cuprizone exposed mice vs controls. After 6 weeks cuprizone exposure, the cuprizone exposed mice has significantly lower s-Ca serum levels than in controls (* p = 0.001). Error bars represent 1 SD. (TIF) [file pone.0026262.s001.tif]

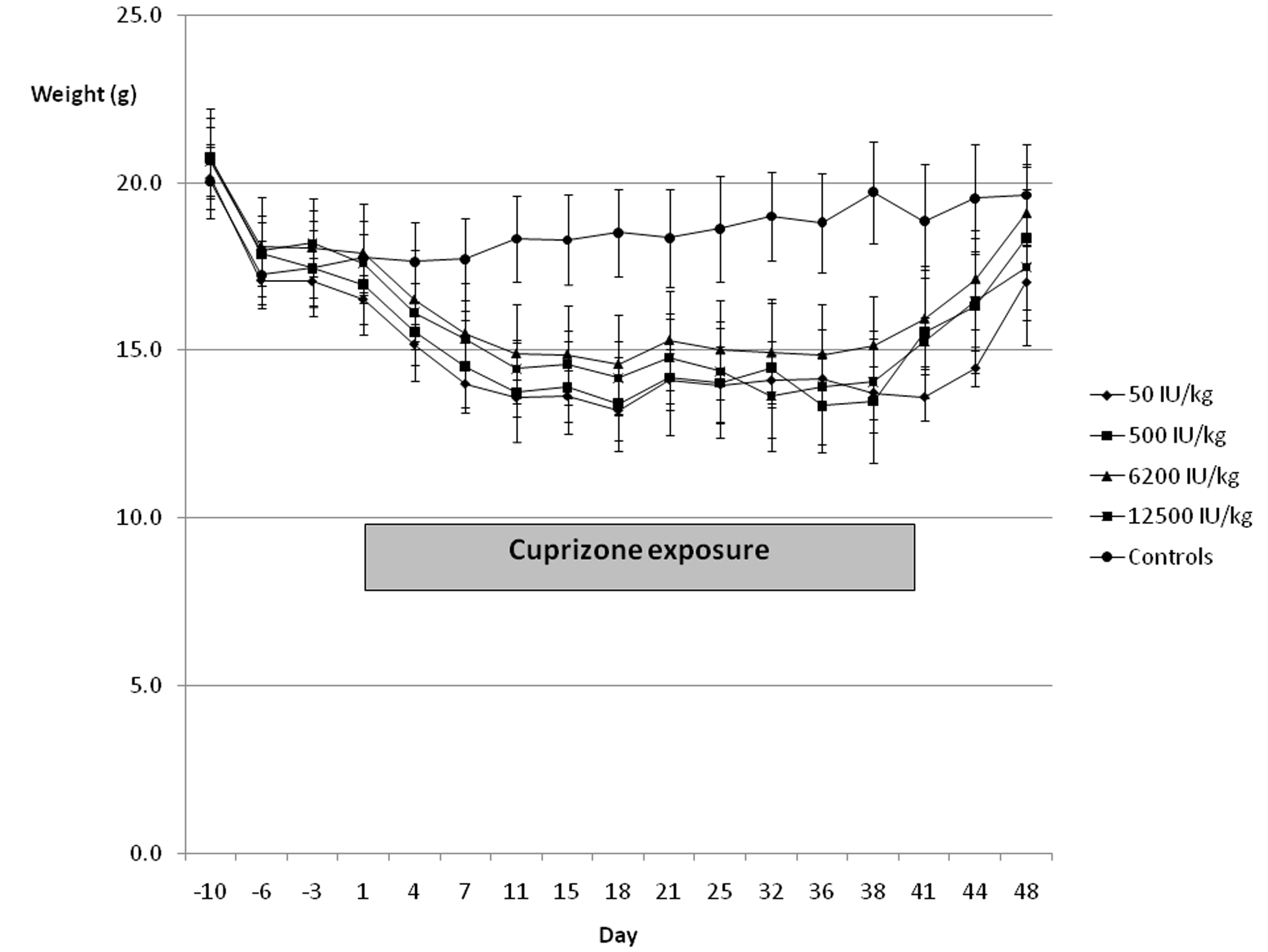

Supplement: Figure S2 — Weight development during the experimental period. Cuprizone exposed mice experienced weight loss compared to the animals in control groups. No differences between the diet groups were detected; neither for cuprizone exposed nor control animals. The control animals are pooled in one group. Error bars represent 1 SD. (TIF) [file pone.0026262.s002.tif]
